# Agreement test of *P* value versus Bayes factor for sample means comparison: analysis of articles from the Angle Orthodontist journal

**DOI:** 10.1186/s12874-023-01858-z

**Published:** 2023-02-16

**Authors:** Natchalee Srimaneekarn, Pattamon Leelachaikul, Sasipa Thiradilok, Somchai Manopatanakul

**Affiliations:** 1 Department of Anatomy, Faculty of Dentistry, Mahidol University, Bangkok, Thailand; 2 Pu-Kradueng Hospital, Pu-Kradueng, Loie, Thailand; 3 Department of Advanced General Dentistry, Faculty of Dentistry, Mahidol University, 6 Yothi Street, Rachtewi, 10400 Bangkok, Thailand

**Keywords:** Agreement test, Bayes factor, *P* value, Effect side, Orthodontics

## Abstract

**Background:**

Researchers are cautioned against misinterpreting the conventional *P* value, especially while implementing the popular t test. Therefore, this study evaluated the agreement between the *P* value and Bayes factor (BF_01_) results obtained from a comparison of sample means in published orthodontic articles.

**Methods:**

Data pooling was undertaken using the modified PRISMA flow diagram. Per the inclusion criteria applied to The Angle Orthodontist journal for a two-year period (November 2016 to September 2018), all articles that utilised the t test for statistical analysis were selected. The agreement was evaluated between the *P* value and Bayes factor set at 0.05 and 1, respectively. The percentage of agreement and Kappa coefficient were calculated. Plotting of effect size against *P* value and BF_01_ was analysed.

**Results:**

From 265 articles, 82 utilised the t test. Of these, only 37 articles met the inclusion criteria. The study identified 793 justifiable t tests (438 independent-sample and 355 dependent-sample t tests) for which the agreement percentage and Kappa coefficient were found to be 93.57% and 0.87, respectively. However, when anecdotal evidence (1/3 < BF_01_ < 3) was considered, almost half of the studies missed statistical significance. Furthermore, two-thirds of the significantly reported *P* values (0.01 < *P* < 0.05; 30 independent-sample and 20 dependent-sample t tests) showed only anecdotal evidence (1/3 < BF_01_ < 1). Moreover, BF_01_ indicated moderate evidence (BF_01_ > 3) for approximately one-third of the total studies, with nonsignificant *P* values (*P* > 0.05). Furthermore, accompanying the *P* values, the effect sizes, especially for studies with independent-sample t tests, were very high with a strong potential to show substantive significance. Although it is best to extend the statistical calculation of a doubted *P* value (just below 0.05), especially for orthodontic innovation, orthodontists may reach a balanced decision relying on cephalometric measurements.

**Conclusions:**

The Kappa coefficient indicated perfect agreement between the two methods. BF_01_ restricted this judgement to approximately half of them, with two-thirds of these studies showing nonsignificant *P* values. Simple extensions of statistical calculations, especially effect size and BF_01,_ can be useful and should be considered when finalising statistical analyses, especially for orthodontic studies without cephalometric analysis.

**Supplementary Information:**

The online version contains supplementary material available at 10.1186/s12874-023-01858-z.

## Background

*Statistics* can be defined as the science of analysing data and drawing conclusions from situations that involve uncertainty. In most cases, uncertainty results from the impossibility or impracticality of studying the entire population [1]. Logically obtaining evidence resulting from the uncertainty of the experiment should depend only on the likelihood principle [2]. Herein, two philosophies of statistical analysis are compared: frequentist and Bayesian. In frequentist statistical inference, the *P* value is the probability of obtaining a test result that is at least as extreme as the observed results of the statistical hypothesis test (particularly in the scope of this study, the t test) assuming the null hypothesis is true [3] (see Eqs. 1 and 2 in the supplemental file). In other words, it is the probability of obtaining a false-positive (Type I error) from the observed data. Fisher originally showed the computation of significance via a continuous quantification of the *P* value. However, hypothesis testing was proposed by Neyman and Pearson [4, 5], where the outcome of a test was based on a dichotomous decision to show evidence in favour of only one hypothesis. The *P* value has gained widespread acceptance for comparison with the significance level (α). This α, set before the study, is the level of the acceptable false-positive rate, while the false negative (Type II error) is minimised [6].

In summary, the *P* value proposed by Fisher, although incompatible with hypothesis testing, was deeply intertwined with the method, as it reveals the probability of errors. Two types of errors exist: a false-positive, which considers two treatment results differently when they are the same (aforementioned as a Type I error or an α error); and a false negative, which considers two treatment results as the same when in fact they are different (Type II error or a β error) [4]. Both the *P* value and hypothesis testing do not measure the evidence but only their statistical significance. Fisher [4] proposed the term ‘significant’ to convey a meaning quite close to the word’s common language interpretation—something worthy of notice. Thus, ‘significant’ is merely worthy of attention in the form of meriting further experimentation but not proof in and of itself [7].

Many studies have warned about the misuse and misinterpretation of the *P* value, emphasising how little information this concept conveys [4, 6–8]. The misinterpretation of the *P* value was documented in the works of psychologists [1], statistical instructors [9], statisticians [10], medicine residents [11], and dentists [12]. Unquestionably, orthodontists are no exception [13]. Although statisticians have long questioned the *P*-value fallacy [14, 15], the t test (with *P* value) and hypothesis testing have been the most popular techniques of inferential statistics used in orthodontic research. Orthodontists routinely perform cephalometric analysis. This analysis comprises approximately 12 to 35 measurements per radiograph. Accordingly, the assessment of treatment results is a comparison of these measurements. In 2017 alone, among the 923 pages of The Angle Orthodontists journal, there were more than 430 t tests. Therefore, for every two pages, one t test (with *P* value) was reported.

Recently, statisticians have signed a petition to bar statistical significance [16–20]. Goodman was concerned about the current lack of evidence-based statistical inference and widespread error in drawing conclusions [6]. He also cited *P* value misconceptions and the possible consequences of this improper understanding [7]. Bayesian estimation was ultimately proposed as a prominent alternative to validate the classical single-number statistical report of the *P* value [10–22].

Bayes’ theorem was first introduced by Thomas Bayes and has been further developed for more than 200 years [23]. Bayes’ theorem is expressed mathematically. (Please see Eqs. 3 and 4 in the supplemental file.)

Bayesian two-sample tests yield slightly improved Type I error rates at the cost of marginally higher Type II error rates. As it does not violate the likelihood principle, Bayesian inference is essentially considered superior to frequentist procedures. Furthermore, the frequentist theory (grounded in average performance) is deemed unrealistic from the Bayesian perspective. Upon scrutiny, Bayesian philosophy provides an abundance of information. The growing number of studies and applications, such as Jeffery’s Bayes factor (BF_01_) and Krushke’s region of practical equivalence (ROPE), shows a promising future [22, 24]. In addition, the Bayes factor on its own quantifies the evidence of statistical judgements in both directions. However, prior evocation clarification is required to bolster impartiality. A detailed comparison has been cogently documented [25].

To provide an alternative to the *P* value, Jeffreys [24] developed the Bayes factor (or, more specifically, BF_10_). It is used to designate the relative strength of evidence for two theories: H_1_ ( alternative hypothesis) and H_0_ (null hypothesis). The subscripts one and zero in BF_10_ indicate the alternative hypothesis over the null hypothesis. In contrast, BF_01_ indicates its inverse ratio (BF_01_ = 1/BF_10_), which is in favour of the null hypothesis. As advocated by Kass and Raferty, the Bayes factor is not just a number that represents the evidential result of one experiment that is often used as a ‘scientific label’ to sway the interpretation to suit the external evidence or author’s belief [26]. It integrates background knowledge (biological understanding and previous research, a priori or often termed the *prior*) into its analysis. The Bayes factor was also supported by Goodman for its uncomplicated interpretability [6]. In this study, ‘Cauchy prior’ to the effect size (δ ~ Cauchy), as described by Rouder and colleagues [21], was calculated. 

While Fisher defined the *P* value as the probability of obtaining a result equal to or more extreme than the observed results of a statistical hypothesis test (under the assumption of no effect or no difference), he also stated that it should be exercised as a nonquantifiable process of drawing conclusions from observations. As previously mentioned, the *P* value does not consider the size of the observed effect. Wasserman attempted to grade the level of interpretation of the *P* value, as shown in Table 1 [27]. However, studies with large sample sizes may yield significant *P* values with only a small effect. Here, effect size comes into play. While the Bayes factor can quantify evidence in favour of the null hypothesis, the *P* value requires an effect size to quantify the magnitude of differences (within the scope of this study). Therefore, exploring these three parameters—*P* value, effect size, and BF_01_—in orthodontic studies is interesting.

**Table 1:** Evidence categorisation for *P* value, effect size, and Bayes factor. Modified Jeffreys’ Bayes factor cutoffs indicating evidence categorised for Bayes factor BF_01_ [27–29]

Measures	Description
*P* value	
< 0.001	Decisive evidence against the null hypothesis
0.001 – 0.01	Substantive evidence against the null hypothesis
0.01 – 0.05	Positive evidence against the null hypothesis
> 0.05	No evidence against the null hypothesis
Effect size	
< 0.2	Small effect size
0.2 – 0.5	Small to medium effect size
0.5 – 0.8	Medium to large effect size
0.8	Large to very large effect size
Bayes factor_01_	
< 1/100	Extreme evidence for the alternative hypothesis
1/30 – 1/100	Very strong evidence for the alternative hypothesis
1/10 – 1/30	Strong evidence for the alternative hypothesis
1/3 – 1/10	Moderate evidence for the alternative hypothesis
1 – 1/3	Anecdotal evidence for the alternative hypothesis
1	No evidence
1 – 3	Anecdotal evidence for the null hypothesis
3 – 10	Moderate evidence for the null hypothesis
10 – 30	Strong evidence for the null hypothesis
30 – 100	Very strong evidence for the null hypothesis
> 100	Extreme evidence for the null hypothesis

## Methods

In recent studies, statisticians have advocated the Bayes factor hypothesis test for t test confirmation. To avoid the misinterpretation fallacy of *P* values, orthodontic researchers can consider using BF_01_ as a quick shortcut to richer (including *prior*) information. Moreover, the prior may facilitate a better understanding of orthodontic research samples and aid in the validation of *P* values. Among the three major orthodontic journals, The Angle Orthodontist journal is the only noncommercial and open-access journal that publishes statistical evaluations of its own articles [30]. Therefore, this study reexamined articles from The Angle Orthodontist journal that used *P* value with a t test by BF_01_. The test agreement and properties of both the *P* value and BF_01_ were evaluated in detail. The effect size was calculated, and its relationships with the *P* value and BF_01_ are shown.

**Fig. 1:**
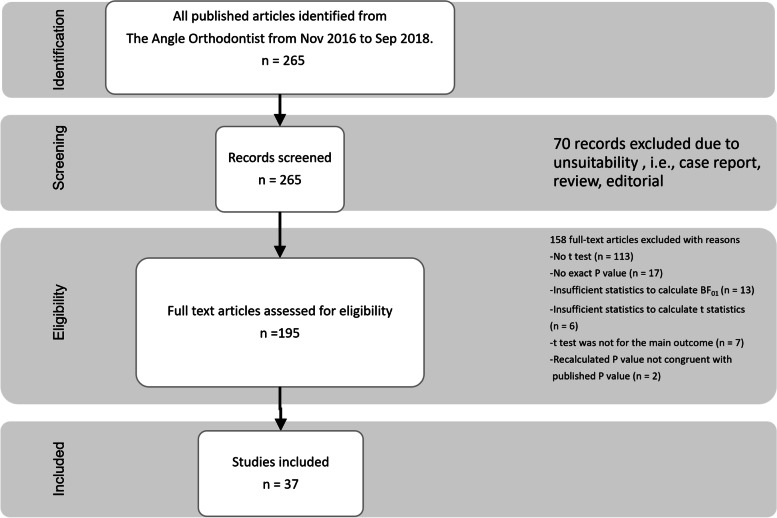
Modified PRISMA 2009 flow diagram showing the process of articles selection

Upon receiving ethical approval from the Institutional Review Board of the Faculty of Dentistry/Faculty of Pharmacy, Mahidol University (MU-DT/PY-IRB 2019/DT014.2703), data pooling was undertaken using the modified Preferred Reporting Items for Systematic Reviews (PRISMA) flow diagram [31]. As per the inclusion criteria applied to the published studies of The Angle Orthodontist journal for a period of two years (November 2016 to September 2018), all articles that utilised the t test for statistical analysis were selected. The exclusion criteria filtered out case reports, editorials, and review papers. Studies that employed statistical analyses other than the t tests were also excluded (Fig. 1).

While the two authors (PL and SM) set the criteria jointly, they independently undertook the tasks of identification, screening, and assessment of the articles’ eligibility for inclusion in the sample. The identified key variables were the mean, standard deviation (SD), *P* value, sample size, and type of t test. All articles that met the requirements of the inclusion criteria, the key variables were evaluated, and the information was input into the pooled data table. The first author (NS), a statistician, then reviewed all articles in the table to resolve any disagreements related to article identification and screening. Parameters from the pooled data table were then used for further analysis, following the steps described below.

### *P* value verification

Initially, the *P* values were recalculated for each study to verify whether they supported the null hypothesis. Studies with incongruent results were excluded from the analysis.

### T-statistic calculation

For the independent-sample t test, an F test was implemented to determine the similarity of variances on a test of ${\mathbb{H}}_{0}: {\sigma }_{1}^{2} = {\sigma }_{2}^{2}$ versus ${\mathbb{H}}_{1}: {\sigma }_{1}^{2} \ne {\sigma }_{2}^{2}$ using the formula as described in the supplemental file (Eq. 6 in the supplemental file) [32]:

Subsequently, the mean, SD, and sample size were input to compute t-statistics using formulae according to the equality (see Eq. 7 in the supplemental file) and inequality (see Eq. 8 in the supplemental file) of the variances.

For the dependent-sample t test, the t-statistic was calculated using the mean, SD, and sample size as inputs into the formula included in the supplemental file (Eqs. 11, 12, and 13 in the supplemental file).

### Computation of *P* value for t-distribution

The calculated t from Eqs. 7 to 13 in the supplemental file with their corresponding degree of freedom (df) were then calculated using the formula shown in the supplemental file (Eq. 14 in the supplemental file) [33].

### BF_01_ calculation

A retest was then carried out using the t-statistic calculated per the aforementioned method (see Eq. 5 in the supplemental file). The computed t-statistic and sample size were then input into R Studio (Package: BayesFactor) [34] using their formula [35]. This formula (see Eq. 5 in the supplemental file) defines the effect size using a default scale setting at 0.707 (δ ~ Cauchy) [3]. To interpret BF_01_, modified Jeffreys’ Bayes factor cutoffs [23, 28] were used in this study. Similarly, the *P* value and effect size were interpreted using the cutoffs given by Wasserman [27] and Cohen [28], respectively. The values of the three cutoffs are shown in Table 1.

### Calculation of effect size

The effect sizes were computed separately for the dependent- and independent-sample t tests. The following formulae denote the employed mathematical calculations for the dependent- (see Eq. 15 in the supplemental file) and independent-sample t tests [36] (see Eq. 16 in the supplemental file).

### Agreement test

The results were then categorised, with the *P* value set to *α* = 0.05 and BF_01_ set to 1. In other words, the results were classified based on whether they rejected the null hypothesis in the condition set. When BF_01_ was greater than 1 and the *P* value was greater than 0.05, it was considered as no evidence against the null hypothesis. However, the null hypothesis was rejected when BF_01_ was less than 1 and the *P* value was less than 0.05. A BF_01_ value of 1 indicated a lack of evidence to either support or reject the null hypothesis. Finally, the agreement percentage and Cohen kappa coefficient were computed.

### Performance evaluation of *P* value, BF_01_, and effect size

Finally, all test and retest results showing the *P* value, BF_01_, and effect size were plotted on a scattergram and analysed in detail.

## Results

**Fig. 2:**
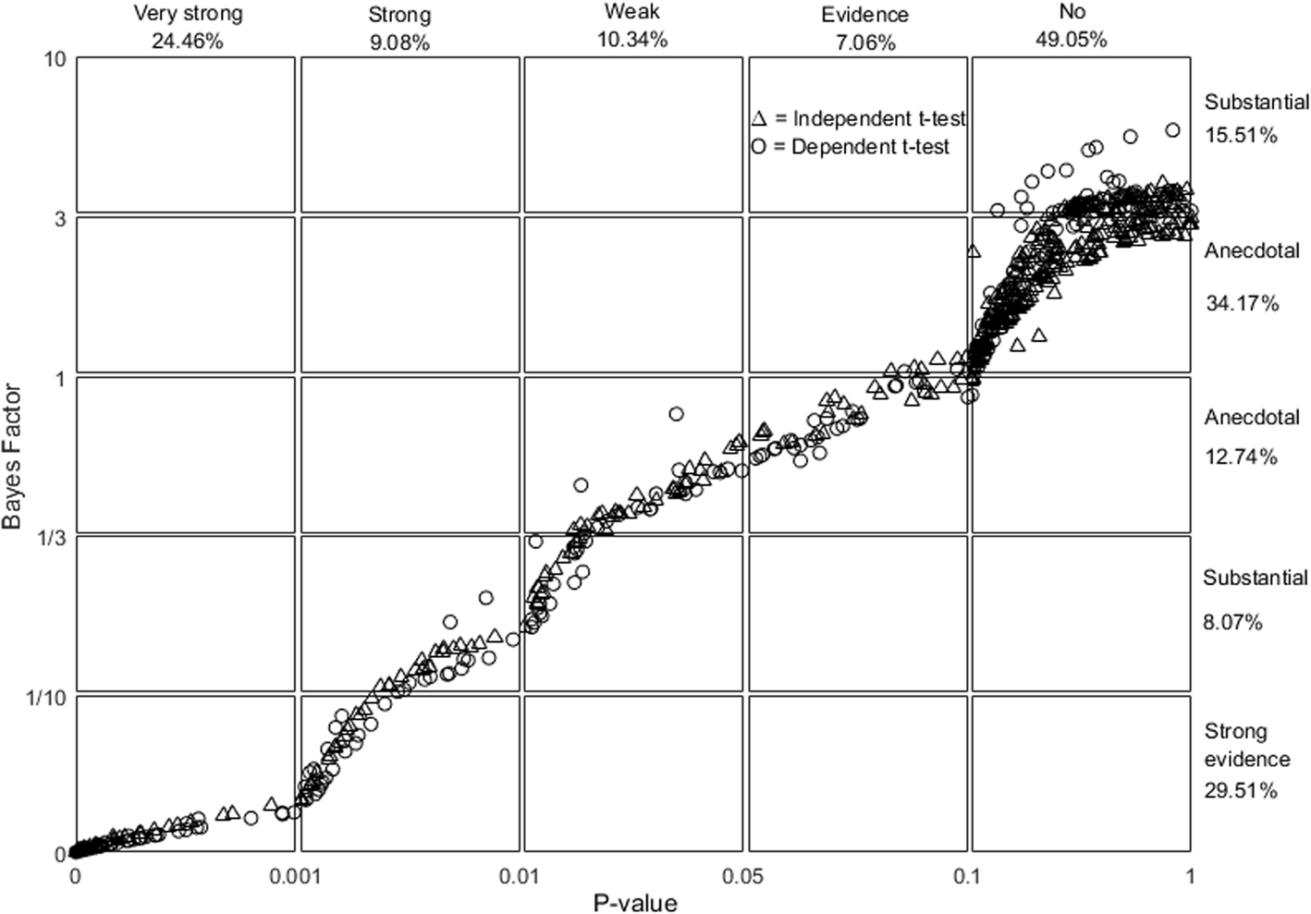
Scattergram of Bayes factor (BF_01_) against *P* value. The triangles denote the independent-sample t test, and circles represent the dependent-sample t test. Some panes are extended to distinguish plot scatter. Accordingly, the scattergram of each pane shows different gradations. This scattergram was created using MATLAB software with a Mahidol University licence

Of 265 articles, 195 showed statistical analyses, and 82 used a student’s t test (42%). Note that only 37 articles satisfied the criteria for the retest method (Fig. 1). The selected articles contained 793 t tests, including 438 independent-sample t tests and 355 dependent-sample t tests. Therefore, when an orthodontic researcher opted for a t test, they reported approximately 21.4 t tests in one article. The evaluation of agreement was performed with the *P* value set at 0.05 and Bayes factor (BF_01_) set at 1. The results showed that most retests (742 retests, 93.57%) produced congruent results. The Cohen kappa coefficient was 0.87, indicating perfect agreement between these two tests. For the studies where the Bayes factor suggested anecdotal evidence (1/3 < BF_01_ < 3) either in favour of or against the effect, most of these studies showed nonsignificant *P* value (322/372 t tests; Table 2). More important, for approximately two-thirds of the reported significant *P* values between 0.01 and 0.05, BF_01_ was between 1/3 and 1 (50/82 t tests). These comprised both types of t tests. The number of independent-sample t tests was approximately 1.5 times that of the dependent-sample t tests (30/20 t tests). Furthermore, BF_01_ quantified the evidence in favour of the null hypothesis, indicating moderate evidence (BF_01_ > 3) for approximately one-third of the total studies with a nonsignificant *P* value (15.51%/56.11%; *P* value > 0.05; Table 2 and Fig. 2).

**Table 2:** Agreement test results

Type of t test	Number of t tests that P indicated evidence						Total
	**BF**_**01**_** only indicated anecdotal evidence**			**BF**_**01**_** could indicate evidence**			
	Nonsignificant *P* value	Significant *P* value	Total	BF_01_ indicated evidence for H_0_ with nonsignificant *P* value	BF_01_ indicated evidence for H_1_, with significant *P* value	Total	
**Independent-sample t test**	232 (29.26%)	30 (3.78%)	262 (33.04%)	65 (8.20%)	111 (14.00%)	176 (22.99%)	438 (55.23%)
**Dependent- sample t test**	90 (11.35%)	20 (2.52%)	110 (13.87%)	58 (7.31%)	187 (23.58%)	245 (30.90%)	355 (44.77%)
**Total**	322 (40.61%)	50 (6.30%)	372 (46.91%)	123 (15.51%)	298 (37.58%)	421 (53.09%)	793 (100%)

*P* value was set to α = 0.05, BF_01_ of 3 to 1/3. Numbers in brackets show percentage of t tests of total studies (793). H_0_: Null hypothesis; H_1_: Alternative hypothesis; BF_01_: Bayes factor_01_

**Fig. 3:**
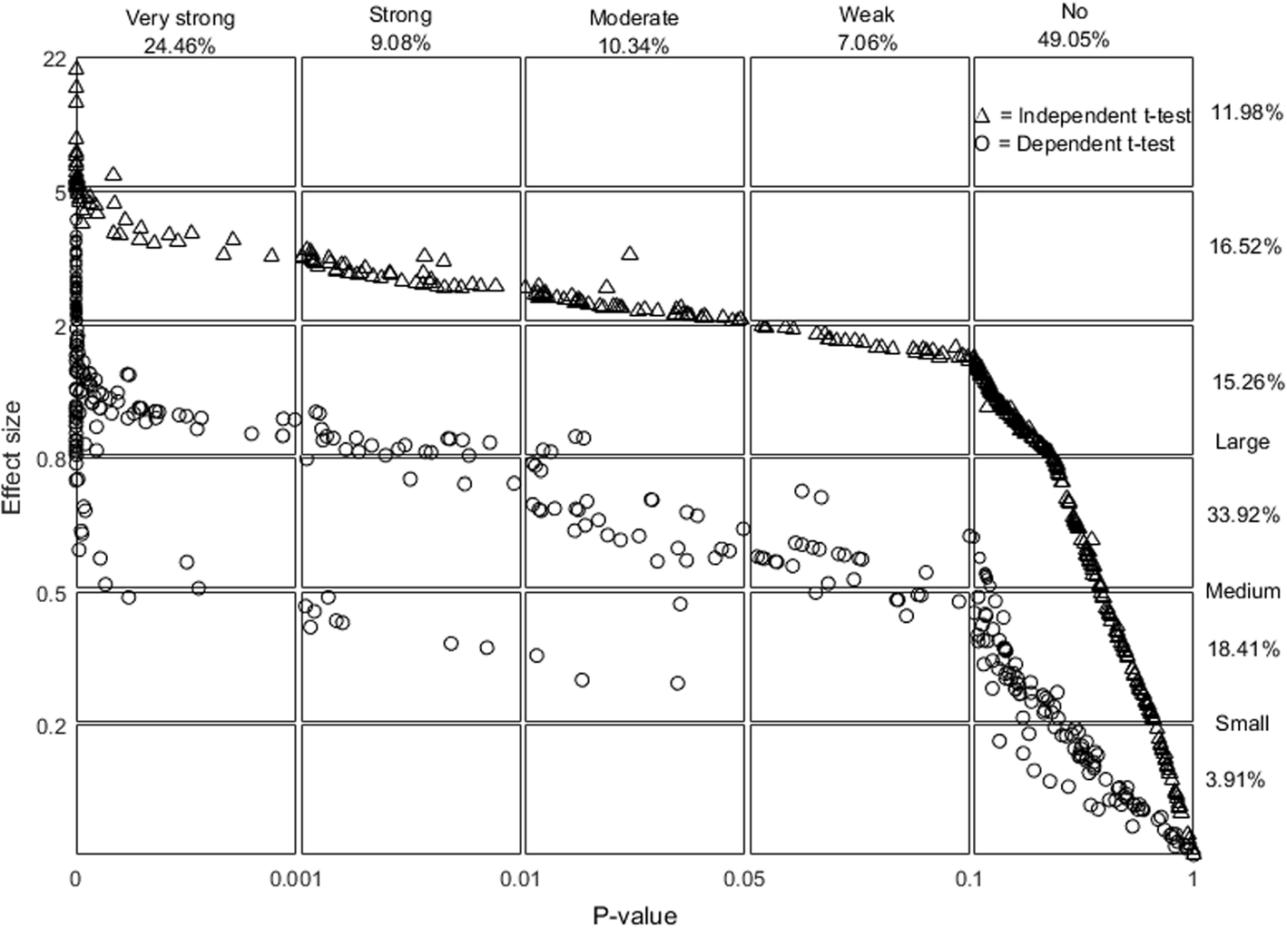
Scattergram of effect size against *P*-value. Plots of the dependent- and independent-sample t tests are observed distinctively. This scattergram was created using MATLAB software with a Mahidol University licence

**Fig. 4:**
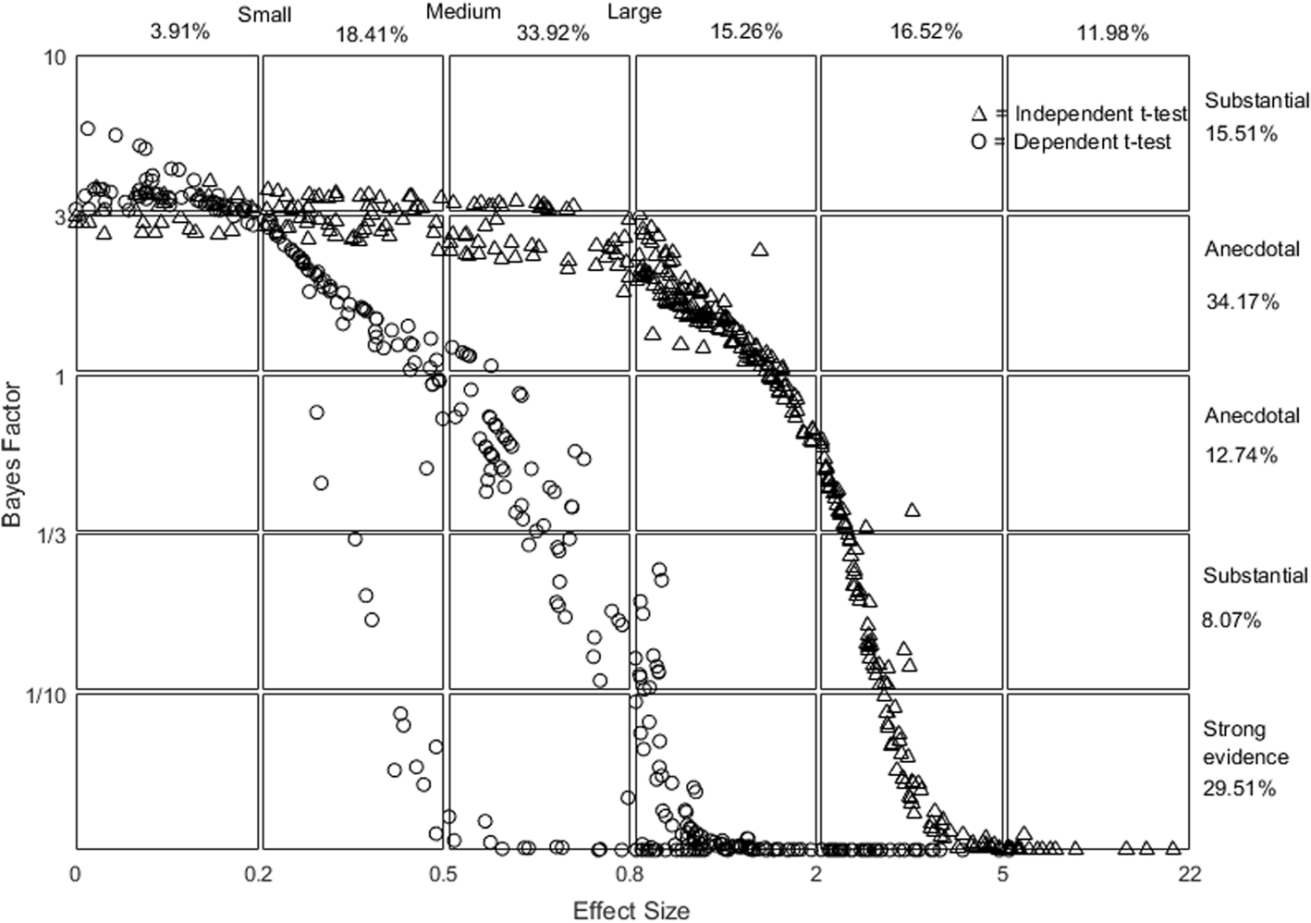
Scattergram of effect size against BF_01_. Distinct plots are observed for the dependent- and independent-sample t tests. This scattergram was created using MATLAB software with a Mahidol University licence

Scattergrams of three parameters—*P* value, effect size, and BF_01_—were plotted and analysed (Figs. 2, 3, and 4). The scale of the axes of all scattergrams mostly followed the categorisation described in Table 1. However, some were extended to distinguish the scatter of the plots. Accordingly, the scattergram of each pane shows different gradations.

Approximately half of the studies showed very large effect sizes (more than 0.8). At the *P* value of 0.05, effect sizes for dependent- and independent-sample t tests were approximately 0.5 and 2, respectively, whereas at the *P* value of 0.01, effect sizes for dependent- and independent-sample t tests were approximately 0.5–1 and 2.5–3, respectively. Moreover, at an effect size of 2, the corresponding *P* values ranged from 0.1 to 0.01, particularly for the independent-sample t test. Interestingly, at an effect size of 0.5, *P* values ranged from 0.5 to 0.001 for the dependent-sample t test. These findings emphasised a moderate relationship between the *P* values and effect sizes but not a strong correlation.

As the decision values of BF_01_ spanned 0.3 < BF_01_ < 3, the interpretation was simpler. The dependent- and independent-sample t test plots of BF_01_ and effect size were still gathered around two separate lines, with a smaller effect size for the dependent-sample t test. The Bayes factor with evidence for the null and alternative hypotheses showed an effect size of a very wide range (0 to 0.8 and 0.5 to 22, respectively).

Considering the three scattergrams, the *P* value and BF_01_ parameters agreed with each other; however, the effect sizes did not. The results began with the extreme side of the decision. The dependent-sample t test revealed a small to medium effect size for the decisive side of no evidence against the null hypothesis as measured by *P* value and decisive evidence for the null hypothesis as measured by BF_01_. The independent-sample t test, by contrast, revealed a small-to-large effect size. More important, on the decisive side of strong evidence against the null hypothesis by *P* value and BF_01_, the dependent-sample t test showed medium to very large effect sizes in most cases. Additionally, the independent-sample t test revealed an extremely large effect size.

Another observation pertained to the *P* values lying on the threshold of a dichotomous decision between the null and alternative hypotheses. While BF_01_ withheld the decision, effect sizes for the dependent-sample t test ranged from low to high. Nevertheless, the independent-sample t test showed very high effect sizes in all cases.

## Discussion

There was agreement on the retest results in most of the included studies. It is well accepted that anecdotal evidence obtained from Bayes factor estimation garners only a bare mention. This study used this area of concern (1/3 < BF_01_ < 3) to evaluate the strength of the *P* value and examine its agreement with BF_01_. When anecdotal evidence was considered (1/3 < BF_01_ < 3), BF_01_ reserved judgement for almost half of the studies. Under the medium Cauchy prior, two-thirds of the frequentist results (0.01 < *P* value < 0.05) would thus be deemed anecdotal evidence (1/3 < BF_01_ < 1) for the alternative hypotheses. Interestingly, in this context of anecdotal evidence, the number of independent-sample t tests was approximately 1.5 times that of the dependent-sample t tests (30/20 t tests). Therefore, further investigation involving the effect sizes using scattergrams is warranted.

One of the most popular advantages of Bayesian statistics is its ability to quantify evidence in favour of the null hypothesis. In this study, BF_01_ provided at least moderate evidence (more than 3) for the null hypothesis in 15.51% of the cases with nonsignificant *P* values. This advantage of BF_01_ over *P* value estimation is crucial and may reduce publication bias, as emphasised by multiple studies [22, 25, 37, 38].

The effect size showed moderate to high agreement but did not agree perfectly with the *P* value and BF_01_. Mostly it facilitated statistical interpretation, as shown in the scatterplot with *P* values and BF_01_. Furthermore, the effect sizes of the selected articles were remarkably large, considering Cohen’s classification [29]. Additionally, the scattergrams showed a distinct separation between the dependent- and independent-sample t test plots. The effect sizes of the dependent-sample t test were mostly smaller than those of the independent-sample t test. Moreover, in the context of the independent-sample t test, when BF_01_ gave the decision for the alternative hypothesis, significant *P* values were reported for two-thirds of the studies. These test results all showed very high effect sizes (greater than 2), complementing the significant *P* values. Orthodontic researchers may be particularly interested in these findings on effect size.

The strength of this study can be derived from the utility of the detailed report on t test parameters published in The Angle Orthodontist journal. First, it should be noted that all t tests from The Angle Orthodontist journal showed standard deviations, and most also displayed exact *P* values. A few studies have indicated additional statistics including confidence intervals, effect sizes, and standard errors. Comprehensive reports on t-test parameters enabled the re-evaluation of the t-test results for the purpose of this study. Second, even though the effect size and BF_01_ calculations are simple, they greatly facilitate statistical interpretation. In addition, to convey the objectivity of the results in orthodontic research, researchers can carry out effect size and BF_01_ estimations. For nonstatisticians, it can be useful to validate statistical interpretation. Furthermore, this one-click away statistical validation is an easy shortcut that requires only a few statistical parameters to make calculations [39–41]. Third, the strength of Bayes’ theorem is that it guides the user toward an understanding of statistical thinking. For nonstatisticians, BF_01_ can serve as a stepping stone to many applications of the Bayes factors. Compared to the frequentist procedure, BF_01_ yields better Type I error control at the expense of an increased Type II error [25]. The practical differences between the varied prior distributions used to calculate BF_01_ are well understood [42]. When the prior is cogently set, BF_01_ provides straightforward and rich information for validation and interpretation. For example, the *prior* incorporated into BF_01_ drives orthodontic researchers to understand the properties of their research samples. For instance, analysing functional magnetic resonance imaging (fMRI), Han recently showed the methodological implication of the adjustment of informative prior distribution that is more suitable to medical imaging studies [43]. Specifically, the adjustment of the parameter prior, the effect size, could be considered for both the centre of the prior distribution to a particular value (depending on theoretical considerations, meta-analysis, or prior research). Moreover, its scale of how wide and narrow the distribution can also be adjusted. Ultimately, it is very challenging for orthodontic researchers to start considering the exclusive prior for orthodontic research and gain these benefits from these innovative and trustworthy studies [43, 44].

Statisticians even recommend that Bayes’ theorem be included in introductory statistics courses as a substitute for inferential statistics [45, 46]. Dienes stated that researchers who foresee what the theory predicts know how much evidence supports the theory [38]. Hence, orthodontic researchers may understand and benefit much more from Bayesian thinking if they determine which evidence from their research supports their hypothesis and their own proposed idea. Moreover, statistical analyses that use Bayesian estimations specifically to mimic t-test evaluations are now widely propounded [21, 46–48]. Basic online Bayes freeware, with a simple interface to allow easy understanding and proper use, has also become available [39–41, 49]. Such software is designed to offer researchers and clinicians more algorithms and parameters to critique before attempting a statistical judgement. These websites offer advanced algorithms and essential parameters that can be used before judging the difference between two sample means. Specifically, Jeffreys's Amazing Statistics Program (JASP) provides a user-friendly stats module with supportive guidance for Bayes factor computation for t test, analysis of variances (ANOVAs), and correlation coefficient without using Bayes factor in Microsoft Excel sheet [41, 50, 51]. These advantages and the easy availability of statistical packages may encourage orthodontists to validate t-test results using the Bayes factor hypothesis test and inspire Bayesian thinking.

This study also has several limitations. First, before the repeated t-test statistical calculations were attempted, an F test was conducted to determine the similarity of variances. Second, during the screening of the articles, a few were found for which multiple t tests were conducted. Thus, the tests were not exactly independent. Third, although studies with analysis of variances were excluded, multiple comparisons of the means offering similar measurements of the same subject might affect the results of this study. Fourth, the retests in this study were set to be very easy by using just the default BF_01_ with a medium Cauchy prior to effect size. (The effect size followed a Cauchy distribution centred on zero with a scale parameter of 0.707 for the alternative hypothesis.) Furthermore, no sensitivity test was conducted in this study. Considering the aforementioned factors, care should be taken when generalising the results of this study or comparing them with those from similar works.

Because of the lack of raw data, additional Bayesian calculations could not be undertaken. This poses another limitation to the present study. To the best of our efforts, BF_01_ was calculated solely from the reported t-test parameters. For future research on the subject, access to raw data sources will be greatly beneficial, allowing published results to be reevaluated using more logical and innovative applications of Bayesian statistics.

### Comparison to similar reports and implications on orthodontic research

Interesting findings lie in the fact that orthodontic studies, while mostly conducted on a small sample size, still show a very high effect. This is important since when the significant *P* value shows the direction, presuming that the treatment effect is present, with this small sample size and high effect side, the significant *P* value from the orthodontic data is more likely to be informative. Second, other fields reported 21% to 31% nonsignificant *P* values [37, 52], and this study showed more nonsignificant *P* values (56.12%). The Bayes factor quantification also revealed that 15.51% showed a BF_01_ of more than 3, indicating moderate evidence for the null hypothesis. Hence, orthodontists might benefit more from Bayes factor quantification than from other fields.

This study emphasises the validation of the t test. Readers can revalidate the summary when encountering the incongruent result of the two parameters, *P* value and Bayes factor, or even with a nonconverging effect size on significant P. It is also crucial to retest the significant P of the innovative orthodontic material or treatment intervention, especially when the *P* value is slightly below 0.05. The supplementary calculation of the effect side and Bayes factor facilitates the analysis of the correctness of the conclusion [50, 51]. From our published research [53], the difference between the ratio of teeth (Cumulative Percentage Ratio7) between Australian and Thai was significantly different (*P* < 0.05). Therefore, it was reasonable to recommend a new ratio to specifically suit Thai patients [one sample t test revealed that t(73) = -2.274; P = 0.029; BF_01_ = 0.7; effect side = 0.378]. At present, scrutinised BF_01_ shows anecdotal evidence by the graphical presentation by JASP summary statistics; it is probably best to restate this conclusion. Furthermore, since this is the only study of its kind and there are no other similar studies, this test should be reconfirmed. Moreover, this simple extension of statistical validation is largely rational, even outside orthodontic research, to dentistry and medical fields.

It should also be mentioned that most orthodontic reports show treatment effects utilising cephalometric analysis. Therefore, the fallacy of only one reported parameter (*P* value) can become less crucial. From the study of soft tissue change by treatment intervention, one parameter was misguided by the *P*-value fallacy [54]. Among all measurements, one of them—the distance from the vertical reference plane (VRP) to Sella-Nasion (Sn) in millimetres—was analysed using a t test, and the misdirected significant P was reported. However, this can be easily verified using freeware equipped with helpful instruction [50, 51], [t(27,28) = -2.102; *P* = 0.040; BF_01_ = 0.61; effect size = 2.107]. Finally, the verification presents one result with an incongruent significant *P* value (although with a large effect size) versus the anecdotal BF_01_. However, since the authors concluded knowledgeably from 25 cephalometric parameters, the final conclusion was not affected by one misdirected *P* value. In short, regarding the orthodontic field armed with cephalometric analysis, it is quite challenging for the *P*-value fallacy to affect the main orthodontic treatment conclusion.

Additionally, familiarity with the Bayes framework has numerous advantages for orthodontic, dental, and medical researchers. First, the Bayes framework is a prominent alternative to following the recommendation of the American Statistical Association (ASA) to go beyond the *P* value [19], especially for orthodontic reports of new treatment interventions or materials. Second, for editors, reviewers, and readers, increasing the sample size to hack the *P* value (data dredging) can be easily verified without knowing the raw data [2, 48, 51]. Finally, it is a self-preparation method for modern Bayes statistical software.

## Conclusions

When comparisons of sample means were retested, the studies published in The Angle Orthodontist journal showed mostly congruent results (Kappa coefficient = 0.87) between the two statistical parameters *P* value and Bayes factor (using the *P* value set at 0.05 and Bayes factor [BF_01_] set at 1). However, when anecdotal evidence (1/3 < BF_01_ < 3) was considered, the Bayes factor reserved judgement for almost half of the studies. However, it quantified moderate evidence favouring the null hypothesis (15.51% nonsignificant *P* values). This advantage of the Bayes factor over the *P* value is crucial for reducing publication bias. In conclusion, statistical judgement should be made with caution. As most clinical orthodontic outcomes were evaluated using cephalometric analysis, drawing conclusions from many cephalometric parameters prevents the P-fallacy effect. Despite this fact, it is recommended that in addition to *P* value computations, other statistical estimations such as effect size or BF_01_ be used to validate judgement and facilitate statistical interpretation. If these parameters show nonconverging results, then the available user-friendly statistical software facilitates this verification. This test is crucial for orthodontic innovative material or treatment modalities.

## Supplementary Information

**Additional file 1.**

## Data Availability

The data supporting this study’s findings are available on The Angle Orthodontists Journal website. (https://meridian.allenpress.com/angle-orthodontist). The dataset analysis of the current study to validate all results is available in the Open Science Framework (OSF) repository, https://osf.io/7rxhg/.

## Abbreviations

α error: Type I error
ANOVAs: Analysis of variances
ASA: American Statistical Association
β error: Type II error
BF: Bayes factor
df: Degree of freedom
fMRI: Functional magnetic resonance imaging
F test: A statistical test based on the F distribution
H_0_: Null hypothesis
H_1_: Alternative hypothesis
JASP: Jeffreys's Amazing Statistics Program
P: Probability
PRISMA: Preferred Reporting Items for Systematic Reviews
SD: Standard deviation
t: Hypothesis test
VRP to Sn: Vertical reference plane to Sella-Nasion
