# Supplementary material for: Agreement test of P value versus Bayes factor for sample means comparison: analysis of articles from the Angle Orthodontist journal
Source: BMC Med Res Methodol. 2023 Feb 16;23:43. doi: 10.1186/s12874-023-01858-z (PMC9933385; doi:10.1186/s12874-023-01858-z)
Supplement: Supplementary file 1 — Additional file 1. [file 12874_2023_1858_MOESM1_ESM.docx]

Supplemental materials

The statistical equations used in this study.

P-value

The P-value is compared with the significance level (α). This α can be described using the power function of a hypothesis test with rejection R, which is the function of θ defined by

$\beta\left( \theta\right):=\mathbb{P}_{\theta}\left( X\in R \right)= \binom{\mathbb{P}\left( Type I Error \right), if \theta\in{}_{0},}{1-\mathbb{P}\left( Type II Error \right), if \theta\in{}_{0}^{c},}$ (1)

A test with power function β(θ) is a level α test if

$\underset{\theta\in{}_{0}}{sup\beta} \left( \theta\right)\leq\alpha,$ (2)

for 0≤α≤1. Hence, the power function is, as a mathematical concept, exactly the object that controls Type I error. With the use of level α tests and the likelihood ratio statistics, it is now possible to choose the c in the rejection region {x:λ(x)≤c}. This warrants the restriction of the size or level α.

Bayes’ theorem

Bayes’ theorem considers a sample space, Ω, which is a set of possible outcomes of a random experiment. An event A is a subset of this sample space or a collection of outcomes. Bayes’ theorem is expressed mathematically as the following equation:

$\mathbb{P}\left( B | A \right)= \frac{\mathbb{P}\left( A | B \right)\mathbb{P(}B)}{\mathbb{P}\left( A \right)}$. (3)

More generally, if B1, B2...Bm form a subset of Ω, then

$\mathbb{P}\left( Bj | A \right)= \frac{\mathbb{P}\left( A | Bj \right)\mathbb{P(}Bj)}{\sum_{i=1}^{m} \mathbb{P}\left( A | Bi \right)\mathbb{P(}Bi)} for any j.$ (4)

Cauchy prior

Cauchy prior to the effect size (δ~Cauchy) was described by Rouder and colleagues^21^. Here, the data x_i_ and y_i_ are distributed as x_i_~*Ɲ*(μ-α/2, σ^2^) and y_i_~Ɲ(μ-α/2, σ^2^), respectively, with grand mean μ, standard deviation σ, and total effect α = δμ. The grand mean μ is assigned a flat prior p(μ) = 1, variance σ^2^ is assigned Jeffreys prior p(σ^2^) =1/ σ^2^, and the effect size δ is assigned a C(0,γ) prior. This prior model is called the Jeffreys-Zellner-Siow (JZS) prior. When used with the t-test for both one and two samples tests, it is called the JZS Bayes factor.

${BF}_{01}$ = $\frac{\left( 1+\frac{t^{2}}{v} \right)^{-(v+1)/2}}{\int_{0}^{\infty} \left( 1+Ng \right)^{-1/2}\left( 1+\frac{t^{2}}{\left( 1+Ng \right)v} \right)^{-(v+1)/2}\left( 2\pi\right)^{-1/2} g^{-3/2}e^{-1/\left( 2g \right)}dg}$, (5)

When ν stands for degree of freedom.

t is the t statistic for the one-sided t test.

N is the number of observations.

$g$ represents Zellner’s g-prior (for a detailed explanation, see references ^1-3^).

$v$ represents degrees of freedom [N (Number of observation)-1].

$e$ is exponential function.

t-statistic calculation for independent samples t-test

For independent samples t-test, the F-test was implemented to determine the similarity of variances on a test of hypothesis $\mathbb{H}_{0}: \sigma_{1}^{2} = \sigma_{2}^{2}$ versus $\mathbb{H}_{1}: \sigma_{1}^{2} \neq\sigma_{2}^{2}$using this formula^4^:

$F= s_{1}^{2} / s_{2}^{2}$ , (6)

where $degree of freedom \left( \nu\right) = n_{1}-1, n_{2}-1$.

If $F>F_{n_{1}-1,n_{2}-1, 1-\alpha/2}$ or $F< F_{n_{1}-1,n_{2}-1, 1- \alpha/2}$,

then $H_{0}$ is rejected.

If $F_{n_{1}-1, n_{2}-1, \alpha/2}\leq F_{n_{1}-1,n_{2}-1, 1- \alpha/2}$

then $H_{0}$ is not rejected, for the α set at 0.05.

Next, the mean, SD, and sample size were input to compute t-statistics using formulae according to the equality (see equation 7) and inequality (see equation 8) of variances, as follow:

$t=$ $\frac{\bar{x}_{1}- \bar{x}_{2}}{s\sqrt{\frac{1}{n_{1}}+ \frac{1}{n_{2}}}}$ , (7)

where $\nu$ = $n_{1}+ n_{2}-2$.

$t=$ $\frac{\bar{x}_{1}- \bar{x}_{2}}{\sqrt{\frac{s_{1}^{2}}{n_{1}}+ \frac{s_{2}^{2}}{n_{2}}}}$ , (8)

where $\nu= \frac{\left( s_{1}^{2}/n_{1}+s_{2}^{2}/n_{2} \right)^{2}}{\left( s_{1}^{2}/n_{1} \right)^{2}/\left( n_{1}-1 \right)+\left( s_{2}^{2}/n_{2} \right)^{2}/\left( n_{2}-1 \right)}$, (9)

and $s= \sqrt{\left[ \left( n_{1}-1 \right)s_{1}^{2}+\left( n_{2}-1 \right)s_{2}^{2} \right]/\left( n_{1}+n_{2}-2 \right)}$ . (10)

t-statistic calculation for dependent samples t-test

For dependent samples t-test, t-statistic was calculated using mean, SD, and sample size as inputs into the following formula:

$\mathcal{t=}\frac{\bar{d}}{\frac{S_{d}}{\sqrt{\mathcal{n}}}}$, (11)

where $\nu= n-1$, $S_{d}= \sqrt{\left[ \sum_{i=1}^{n} d_{1}^{2}- \left( \sum_{i=1}^{n} d_{1} \right)^{2}/ n \right] / \left( n-1 \right)}$ . (12)

and $S_{d}= \sqrt{\left[ \sum_{i=1}^{n} d_{1}^{2}- \left( \sum_{i=1}^{n} d_{1} \right)^{2}/ n \right] / \left( n-1 \right)}$ . (13)

Computation of the P-value for t distribution

Calculated t from equations 7, 8 and 10 with their corresponding df were then calculated using this following formula^5^:

$p=2\int_{\left| t \right|}^{\infty} \frac{\Gamma\left( \frac{v+1}{2} \right)}{\sqrt{v\pi} \Gamma\left( \frac{v}{2} \right)} \left( 1+\frac{t^{2}}{v} \right)^{-\left( v+1)/2 \right)}dt$, (14)

where $\Gamma$ is the gamma function.

Computation of effect size

The effect sizes (d) were computed separately for the dependent and independent samples t-tests. The following formulae denote the employed mathematical calculations respectively for the dependent (see equation 15) and independent samples t-tests (see equation 16)^6^:

$\mathcal{d=}\frac{M_{\mathcal{d}}}{S_{d}}$ , (15)

$\mathcal{d=}\frac{M_{e}-M_{\mathcal{c}}}{S_{pooled}}$ . (16)

Md stands for mean difference.

Sd is standard deviation of the difference.

Me represents mean of the experimental group.

Mc represents mean of control group.

Spooled stands for pooled standard deviation.

References

1. Liang F, Paulo R, Molina G, et al. Mixtures of g priors for Bayesian variable selection. JASA. 2008;103:410-23.
2. Zellner, A. On assessing prior distributions and Bayesian regression analysis with g-prior distributions. In Goel PK, Zellner A (Eds.), Bayesian inference and decision techniques: Essays in honor of Bruno de Finetti. Amsterdam: North-Holland;1986.
3. Zellner A, Siow A. Posterior odds ratios for selected regression hypotheses. In Bernardo JM, DeGroot MH, Lindley DV, et al. (Eds.), Bayesian statistics. Valencia: Valencia University Press;1980.
4. Rosner B. Fundamentals of Biostatistics 8^th^ ed. Boston, MA: Cengage Learning; 2015.
5. Krishnamoorthy K. Handbook of statistical distributions with applications. Boca Raton, FL: Chapman and Hall; 2006.
6. Mussweiler T. Doing is for thinking! Psychol Sci. 2006;17:17-21.https://doi.org/10.1111/j.14679280.2005.01659.x.
